# Novel and diverse mycoviruses co-infecting a single strain of the phytopathogenic fungus *Alternaria dianthicola*

**DOI:** 10.3389/fcimb.2022.980970

**Published:** 2022-09-27

**Authors:** Jie Zhong, Ping Li, Bi Da Gao, Shuang Yu Zhong, Xiao Gang Li, Zhao Hu, Jun Zi Zhu

**Affiliations:** ^1^ Hunan Engineering Research Center of Agricultural Pest Early Warning and Control, Hunan Agricultural University, Changsha City, China; ^2^ Hunan Provincial Key Laboratory for Biology and Control of Plant Diseases and Insect Pests, Hunan Agricultural University, Changsha City, China

**Keywords:** mycoviruses, *Alternaria dianthicola*, virome, virus diversity, biocontrol

## Abstract

*Alternaria dianthicola* is a pathogenic fungus that causes serious leaf or flower blight on some medicinal plants worldwide. In this study, multiple dsRNA bands in the range of 1.2-10 kbp were found in a *Alternaria dianthus* strain HNSZ-1, and eleven full-length cDNA sequences of these dsRNA were obtained by high-throughput sequencing, RT-PCR detection and conventional Sanger sequencing. Homology search and phylogenetic analyses indicated that the strain HNSZ-1 was infected by at least nine mycoviruses. Among the nine, five viruses were confirmed to represent novel viruses in the families *Hypoviridae*, *Totiviridae*, *Mymonaviridae* and a provisional family Ambiguiviridae. Virus elimination and horizontal transmission indicated that the (-) ssRNA virus, AdNSRV1, might be associated with the slow growth and irregular colony phenotype of the host fungus. As far as we know, this is the first report for virome characterization of *A. dianthus*, which might provide important insights for screening of mycovirus for biological control and for studying of the interactions between viruses or viruses and their host.

## Highlights

1. This research reported the characterization of the virome of an Alternaria dianthicola strain HNSZ-1.
2. Among the nine co-infecting viruses, five were novel viruses belonging to families *Hypoviridae, Totiviridae, Mymonaviridae* and a provisional family Ambiguiviridae.
3. Virus elimination, horizontal transmission and biological comparison revealed that the (-) ssRNA virus, AdNSRV1, might be related with the phenotypic change of the host fungus.

## Introduction

Mycoviruses (fungal viruses) are commonly infect all major groups of fungi, including plant-pathogenic fungi (Ghabrial and Suzuki, 2009; Pearson et al., 2009). In general, mycovirus infections are cryptic, while some other fungal viruses were associated with hypovirulence which can alter several physiological and biochemical properties of their fungal hosts (Nuss, 2005; Pearson et al., 2009; Yu et al., 2010; Yang et al., 2018). Hypovirulence-associated mycoviruses have the potential to be used as a biocontrol material to control fungal diseases. The successful use of Cryphonectria hypovirus 1 (CHV1) to control chestnut blight in Europe has pioneered the use of mycoviruses for biocontrol (Rigling and Prospero, 2018). Recently, the DNA mycovirus Sclerotinia sclerotiorum hypovirulence associated DNA virus 1 (SsHADV-1) has been proven to be a usefully biocontrol agent since it could be transmitted extracellularly and convert the pathogenic host fungus into a beneficial, non-pathogenic endophyte (Rigling and Prospero, 2018; Zhang et al., 2020). Therefore, hunting of mycoviruses conferring hypovirulence has become a pathway for development of potential biological control strategies for fungal diseases (Xie and Jiang, 2014; Ghabrial et al., 2015; García-Pedrajas et al., 2019).

Recently, with the development of high throughput sequencing, more and more studies revealed mixed infection of different mycoviruses or dsRNA elements in a single strain, such as the fungal strains *Rosellinia necatrix*, *Botrytis cinerea*, *Rhizoctonia solani*, *Sclerotinia sclerotiorum*, *Sclerotium rolfsii* (Xie and Ghabrial, 2012; Kondo et al., 2013; Bartholomäus et al., 2016; Hao et al., 2018; Zhu et al., 2018). For example, 17 different mycoviruses have been reported to co-infect a *Rhizoctonia solani* strain DC17 (Bartholomäus et al., 2016), and 21 mycoviruses have been found in a hypovirulent *Sclerotium rolfsii* strain BLH-1 (Zhu et al., 2018). Mu et al. (2021) have also reported 9 mycoviruses that were assigned into eight potential families and exhibited new evolutionary modes in a single *S. sclerotiorum* strain SX276. Different mycoviruses co-infected in a single fungal strain might provide some possibilities for virus recombination or horizontal gene transfer, thus promoting the evolution of viruses (Vijaykrishna et al., 2015). In addition, co-infections by different mycoviruses might also provide theories for virus classification and interaction. It is known that some synergistic or antagonistic interactions might exist between co-infected mycoviruses, that might enhance or decrease their disease symptoms (Hillman et al., 2018; Thapa and Roossinck, 2019). Recently, there are examples of virus interactions between capsidless (+) ssRNA and dsRNA virus, where the former which belong mainly to *Yadokariviridae* can hijack the latter’s particles, such as the viruses in the order *Ghabrivirales* or genus *Botybirnavirus*, for packaging (Zhang et al., 2016; Das et al., 2021; Jia et al., 2022).

*Alternaria* sp. are notorious pathogens which are widely distributed in the natural environment and can cause disease in a wide variety of organisms including plants, humans and animals. Some of the phytopathogenic species can infect a wide range of plant species causing major losses for a large number of crops (Woudenberg et al., 2013). Moreover, toxins produced by some *Alternaria* species accumulated in agricultural products would also pose a threat to food safety (Ambra et al., 2013). *Alternaria dianthcola* is a plant pathogen that can cause flower blight on carnation in China and leaf blight on *Withania somnifera* in India, that brought great damage to such medical and ornamental plants (Maiti et al., 2007; Duan et al., 2015). Up to now, many mycoviruses have been reported in *Alternaria* spp., and some of them are related with the change of host phenotype. For example, Alternaria alternata hypovirus 1 (AaHV1) that was isolated from *Alternaria alternata* in an apple orchard has been confirmed to confers hypovirulence in its host and other plant phytogenic fungi (Li et al., 2019). Alternaria alternata chrysovirus 1 (AaCV1) has been described to reduce growth rate of host fungus, but enhances the pathogenicity (Okada et al., 2018). In addition, AaCV1-AT1 has also been reported to reduce colony growth rate and conidial production ability on its host fungus (Ma et al., 2020). These studies indicated that there are many mycoviruses that might possess biocontrol potential in the fungal strains of *Alternaria* sp.

In this study, we molecularly characterized a variety of different mycoviruses from a single *A. dianthcola* strain HNSZ-1, by high-throughput sequencing, RT-PCR detection and conventional Sanger sequencing. In addition, effects of these viruses on their host were also been discussed.

## Materials and methods

### Fungal isolates and growth conditions

*A. dianthcola* strain HNSZ-1 was collected from diseased leaf of *Dianthus chinensis* in Hunan province of China. All the strains were cultured on potato dextrose agar (PDA) at 27°C. The long-term preservation of fungal isolates was conducted by picking fungus plugs, growing on PDA for 7-10 days, in glycerin at -80°C. For dsRNA extraction, mycelial plugs were cultured on PD, with reciprocal shaking of 180 rpm, for 5 days at 27°C. Mycelia were harvested by filtration with sterile gauze.

### DsRNA extraction and high-throughput sequencing

Extraction of dsRNA from mycelia was conducted by cellulose chromatography using the method as described by Morris and Dodds (Morris and Dodds, 1979). The dsRNA extractions were treated by DNase I or S1 nuclease (TaKaRa, Dalian, China) to eliminate possible contaminated DNA and ssRNA. The dsRNAs were then analyzed by 1% agarose gel electrophoresis.

The cDNA library construction and high-throughput sequencing was prepared using the previously described protocol (Bartholomäus et al., 2016). The cDNA was synthesized with the universal Primer-dN6 (GCCGGAGCTCTGCAGAATTC NNNNNN), and the dscDNA was amplified using the Primer (GCCGGAGCTCTG CAGAATTC). All the PCR products were purified, sheared, and ligated with adaptors for Illumina sequencing. Sequencing was performed on the Illumina MiSeq 2000/2500 system by a pair-end sequencing run.

### Bioinformatics analyses

Deep sequencing data was analyzed by CLC Genomic Workbench software package (CLC Bio-Qiagen, Boston, MA). After removing the low-quality sequence at the end and the joint sequence, the Clean Data was obtained and *de novo* assembled using the Trinity software (Grabherr et al., 2011). All generated contigs were compared to the National Center for Biotechnology Information (NCBI) database using BLASTx or tBLASTx with standard settings. The viral sequences were picked out and subjected for further confirmation.

### Viral genome sequences validation and their full-length determination

To verify the presence of putative viruses in strain HNSZ-1, RT-PCR detections were conducted using specific primers designed according to the viral sequences obtained from the Illumina sequencing. Total RNA was isolated from collected mycelial mass using the RNeasy mini kit (Qigen, Valentia, CA). The cDNA sequences were generated by reverse transcription reaction using RevertAid First Strand cDNA Synthesis Kit and hexdeoxyribonucleotide mixture random primers (Takara Dalian, China). PCR amplifications were carried out using the cDNA as temple and the amplicons were analyzed by agarose gel electrophoresis and sequenced.

In order to complete the obtained viral genome sequences, the sequence gaps were filled by RT-PCR amplification with specific primers, and the 5’-and 3’-terminal sequences of all viral sequences infecting strain HNSZ-1 were obtained by ligase-mediated terminal amplification method as described previously (Xiang et al., 2017). The expected PCR amplicons were purified and cloned into pMD18-T vector (TaKaRa) for sequencing, with each base being sequenced in at least three independent clones.

### Mycoviral genome analysis and phylogenetic analysis

Homology searches were conducted using the BLAST (BLASTp or BLASTx) program in NCBI. Potential open reading frames (ORFs) were predicted using the online NCBI ORF Finder tool. Multiple sequence alignments were performed using ClustalX program (Thompson et al., 1997). In order to determine the taxonomic status of the identified viruses, phylogenetic trees were constructed based on the amino acid sequence alignments of RNA-dependent RNA polymerase (RdRp) regions by neighbor-joining (NJ) method in MEGA 6, with bootstrap values calculated by of 1,000 replicates (Tamura et al., 2013).

### Protoplast preparation, virus horizontal transmission and biological test

In order to clarify the biological effects of these viruses on HNSZ-1 strain, virus-curing by protoplast regeneration was conducted using the previously described method (Lau et al., 2018). Single-protoplast regeneration derivatives were selected and cultured at PDA for 7-10 days. Hyphal anastomosis was carried out for virus horizontal transmission by co-culturing the donor and recipient strains in a single PDA plate as be described previously (Li et al., 2019). The presence of individual viruses was tested by dsRNA extraction and RT-PCR detection, using specific primers based on the RdRp-encoded sequences. For biological test, agar plugs of fresh mycelia picked from the colony margins of actively grown cultures were placed on fresh PDA plates and cultured at 27°C. Then, the colony diameter of each strain was measured, and colony morphology was examined. Experiments were performed at least twice, with each treatment being conducted by three replicates. The data for growth rates were analyzed by one-way analysis of variance using the SPASS software. Differences with P values of <0.05 were considered statistically significant.

## Results and discussion

### Multiple dsRNA species in strain HNSZ-1

When extracted by cellulose chromatography and digested with DNase I and S1 nuclease, diverse dsRNAs fragments were detected from strain HNSZ-1, as illustrated using agarose gel electrophoresis, which indicated the presence of multiple mycoviruses infection. At least eight dsRNA fragments, ranging from 1.2 kbp to 10 kbp in size, were observed (**Figure 7A**).

To investigate the mycoviruses infecting strain HNSZ-1, high-throughput sequencing library was constructed and sequenced on Illumina MiSeq 2500 platform. A total of 34,478,052 raw reads were obtained. After filtering of the low-quality reads, 34,315,594 high-quality sequence reads were *de novo* assembled into 5,924 contigs. Through preliminary BLASTx against to the NCBI NR database, 11 assembled contigs similar to viruses were obtained. Among these contigs, 9 contigs were significantly similar to the amino acid sequences of RdRp domains of 9 different viral sequences, suggesting that these contigs represent 9 distinct nearly complete mycovirus-like genome sequences. Analysis of the putative viral sequences indicated that they were belonged to +ssRNA mycoviruses in the families *Narnaviridae*, *Hypoviridae*, *Ambiguiviridae*, -ssRNA mycovirus in the family *Mymonaviridae*, and dsRNA mycoviruses in the families *Totiviridae*, *Partitiviridae* and an unclassified taxon. To identify the really presence of these mycoviruses in strain HNSZ-1, RT-PCR was performed using the total RNA extracted from strain HNSZ-1 and specific primers designed based on the viral contigs. Results showed that all the viral contigs were probable viral origin.

The terminal genome sequences of the mycoviruses were determined by using ligase mediated terminal amplification technique. In combination with the high-throughput sequencing and conventional Sanger sequencing, the genome sequences of the 9 different mycoviruses were completed. Detailed information of these mycoviruses including their best matches deduced by BLASTx searches were listed in **Table 1**. According to the subsequent sequence analysis, those mycoviruses were temporarily designed as Alternaria dianthicola hypovirus 1 (AdHV1), Alternaria dianthicola narnavirus 1 (AdNV1), Alternaria dianthicola narnavirus 2 (AdNV2), Alternaria dianthicola umbra-like virus 1 and 2 (AdULV1), Alternaria dianthicola umbra-like virus 2 (AdULV2), Alternaria dianthicola victorivirus 1 (AdVV1), Alternaria dianthicola partitivirus 1 (AdPV1), Alternaria dianthicola negative-stranded RNA virus 1 (AdNSRV1), Alternaria dianthicola dsRNA virus 1 (AdRV1). Among the identified viruses, AdRV1 was reported previously in our lab (Hu et al., 2020), thus, we focused on other eight viruses in the following analysis.

**Table 1:** Analysis on viral sequences discovered in the strain HNSZ-1.

Virus name	Sequence length (bp)	Closest relative virus	Maximum Identity (%)	Genome type	Tentative virus classification	Accession numbers
Alternaria dianthicola hypovirus 1	9443	Fusarium concentricum hypovirus 1	58.82	+ssRNA	*Hypoviridae*	ON843755
Alternaria dianthicola narnavirus 1	3159	Erysiphe necator associated narnavirus 48	96.74	+ssRNA	*Narnaviridae*	ON843756
Alternaria dianthicola narnavirus 2	2192	Plasmopara viticola lesion associated narnavirus 1	97.09	+ssRNA	*Narnaviridae*	ON843757
Alternaria dianthicola umbra-like virus 1	4568	Plasmopara viticola lesion associated ambiguivirus 1	54.49	+ssRNA	*Ambiguiviridae*	ON843758
Alternaria dianthicola umbra-like virus 2	3380	Macrophomina phaseolina umbra-like virus 1	48.09	+ssRNA	*Ambiguiviridae*	ON843759
Alternaria dianthicola victorivirus 1	5318	Fusarium asiaticum victorivirus 2	58.50	dsRNA	*Totiviridae*	ON843760
Alternaria dianthicola partitivirus 1	S1:1770 S2:1553 S3:1297	Alternaria tenuissima partitivirus 1	94.26	dsRNA	*Partitiviridae*	ON843761 ON843762 ON843763
Alternaria dianthicola negative-stranded RNA virus 1	9013	Sclerotinia sclerotiorum negative-stranded RNA virus 3-U	56.03	-ssRNA	*Mymonaviridae*	ON843764
Alternaria dianthicola dsRNA virus 1	3014	Trichoderma harzianum mycovirus 1	68.89	dsRNA	Unclassified	MT241326 (Hu et al., 2020)

### One novel virus characterized in the family *Hypoviridae*

Viruses in the family *Hypoviridae* are +ssRNA viruses whose genomes generally ranged from 9 to 13 kbp containing one or two ORFs (Ghabrial et al., 2015; Suzuki et al., 2018). In the past, *Hypoviridae* contained only one genus, *Hypovirus*, including the mycoviruses isolated from *Cryphonectria parasitica.* At present, the members of this family are divided into three genera, including *Alphahypovirus*, *Betahypovirus* and *Gammahypovirus*. Viruses belong to *Alphahypovirus* were represented by Cryphonectria hypovirus 1 (CHV1) and Cryphonectria hypovirus 2 (CHV2), which contained two ORFs and in a larger size (12.5-13 kbp) than those in betahypovirus (9.1-10.4 kbp). Genus *Betahypovirus* was represented by CHV3 and CHV4 encoding only one polyprotein and four conserved protein domains. Viruses in the Genus *Gamahypovirus* contained the largest genome represented by Sclerotinia sclerotiorum hypovirus 2 (Mahmoud and Michael, 2014). While Setosphaeria turcica hypovirus 1 (StHV1) from *Setosphaeria turcica*, Valsa ceratosperma hypovirus 1 (VcHV1) from *Valsa ceratosperma* (Hajime et al., 2012), CHV3 and CHV4 from *Cryphonectria parasitica* are supposed to be part of the *Betahypovirus* genus, encoding only one polyprotein and four conserved protein domains. Viruses in the *Gammahypovirus* genus were larger than other viruses and only contained a single ORF.

**Figure 1:**
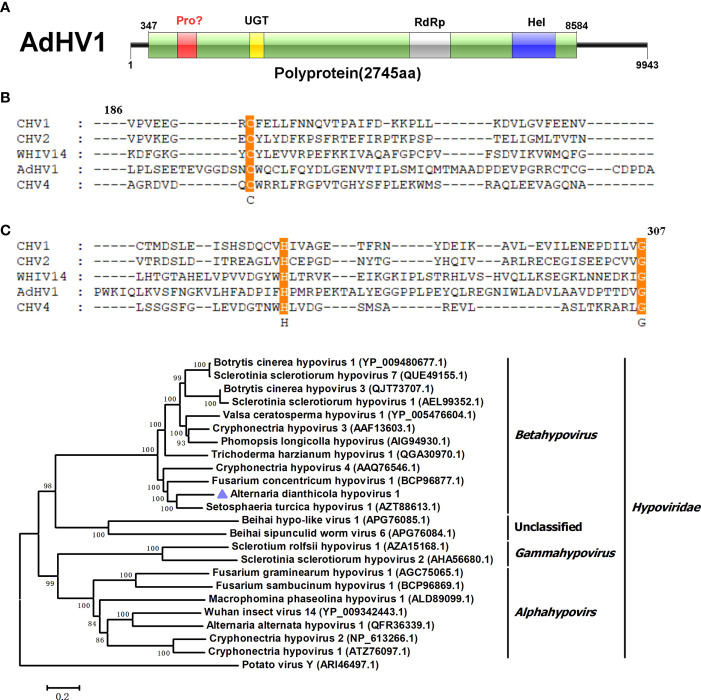
Genome properties of Alternaria dianthicola hypovirus 1 (AdHV1). **(A)** Schematic representation of the AdHV1 genome. The open reading frame (ORF) was shown as box, and the aa size of the encoded polyprotein was indicated. The color squares indicated the position of domains of the predicted polyprotein, including UGT (UDP-glucosyltransferase), RdRp (RNA-dependent RNA polymerase), and Hel (helicase-associated domain). **(B)** Alignment of the amino acid sequence of the regions corresponding to cysteine protease between AdHV1 and other hypoviruses. The conserved three cysteine protease core residues, cysteine, histidine, and glycine were highlighted. **(C)** Phylogenetic tree based on the RdRp domains constructed by neighbor-joining (NJ) method in MEGA 6 with a bootstrap value of 1,000 replicates. The numbers at the branches indicated the bootstrap values supporting the branches. The scale bar at the lower represented a genetic distance of 0.2. AdHV1 was highlighted in the phylogenetic tree.

In this study, a 9,443 bp long RNA fragment with a G+C content of 48.77% was identified, which showed similarity to viruses in the *Hypoviridae* family and be designated as AdHV1. AdHV1 contains a large ORF, encoding a 2,745 amino acid polyprotein with a calculated molecular mass of 310.17 kDa (**Figure 1A**). Three conserved domains of UDP-glucosyltransferase, RdRp and helicase-associated domain were found. It is well known that many polyproteins encoded by hypoviruses, such as CHV1 and CHV4, were processed by papain-like cysteine protease that was located in the N-terminal regions (Koonin et al., 1991; Aulia et al., 2021). Unexpectedly, the papain-like cysteine protease motif was not found in the AdHV1 polyprotein by conserved domain search in NCBI. However, alignment of the N-terminal region of the AdHV1 (186-307 aa region) with the papain-like cysteine protease regions of other hypoviruses revealed the presence of conserved three cysteine protease core residues, including cysteine, histidine and glycine (**Figure 1B**). This indicated that the AdHV1 encoded polyprotein might also be processed by cysteine protease. BLASTp homology search revealed that the polyprotein of AdHV1 had 58.63% to 67.61% aa sequence identity to proteins encoded by viruses in genus *Betahypovirus*, including Setosphaeria turcica hypovirus 1 (StHV1), Fusarium concentricum hypovirus 1 (FcHV1) and CHV4 (Daniela et al., 2005; Gilbert et al., 2019; Mizutani et al., 2021). Phylogenetic tree of AdHV1 and other related hypoviruses were generated using the conserved regions of polyprotein, it revealed that AdHV1 is a novel member of the family *Hypoviridae* (**Figure 1C**).

### Two viruses in the family *Narnaviridae*

**Figure 2:**
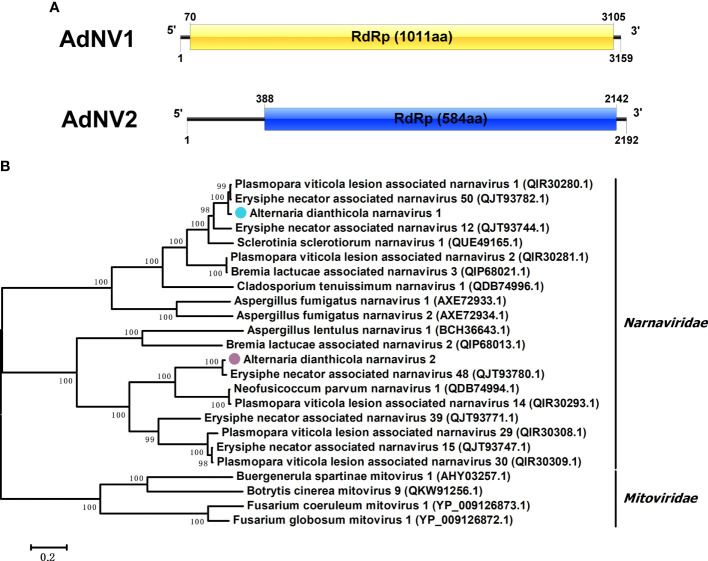
Genome organization and phylogenetic tree of Alternaria dianthicola narnavirus 1 and 2 (AdNV1 and AdNV2). **(A)** Schematic diagram of the genome organizations of AdNV1 and AdNV2. Each of the AdNV1 and AdNV2 genomes contained a single ORF encoding the RdRp as showed by the rectangular boxes. **(B)** A NJ phylogenetic tree utilizing the RdRps of AdNV1, AdNV2 and other viruses in the families *Narnaviridae* and *Mitoviridae.* The numbers at the branches indicated the bootstrap values, and the bars corresponded to the genetic distance.

Viruses in the *Narnaviridae* family contained the simplest genomes, ranging from 2.3 to 3.6 kbp, and included a single ORF encoding RdRp (Hillman and Cai, 2013). Two contigs showed similarity to members of the family *Narnaviridae*. We designated the two viruses as AdNV1 and AdNV2. AdNV1 and AdNV2 were in lengths of 3159 and 2192 nucleotides (nts), respectively (**Figure 2A**). Like most narnaviruses, both the AdNV1 and AdNV2 contained a single ORF, encoding proteins that showed 96.74% and 97.09% identities to the RdRps of Erysiphe necator associated narnavirus 48 and Plasmopara viticola lesion associated narnavirus 1, respectively (Chiapello et al., 2020). Phylogenetic analysis indicated that AdNV1 and AdNV2 were grouped in the clade of *Narnavirus* genus, in the family *Narnaviridae* (**Figure 2B**).

### Two novel viruses in the family *Ambiguiviridae*

**Figure 3:**
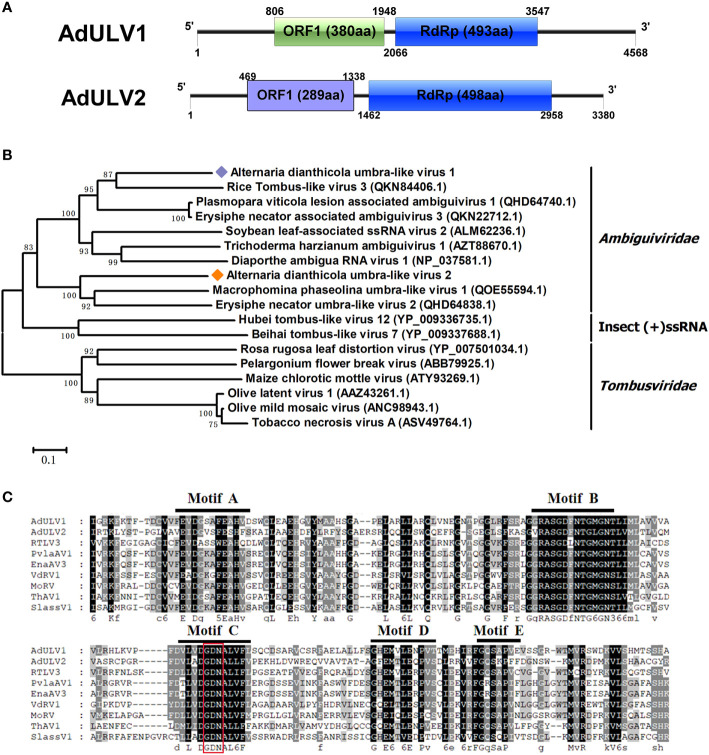
Genomic architecture and phylogenetic analysis of Alternaria dianthicola umbra-like virus 1 and 2 (AdULV1 and AdULV2). **(A)** Schematic representation of AdULV1 and AdULV2. Each the genomes of AdULV1 and AdULV2 possessed two ORFs, encoding a hypothetical protein and a RdRP, respectively. **(B)** Phylogenetic analysis using the RdRp aa sequences of AdULV1 and AdULV2, other selected members of the proposed family Ambiguiviridae and the known family *Tombusviridae*. The bootstrap values were calculated by 1,000 replicates. AdULV1 and AdULV2 were indicated in the phylogenetic tree. **(C)** Multiple aa sequences alignment of the RdRp of AdULV1 and AdULV2 and other related viruses in the proposed family Ambiguiviridae. The conserved motifs were indicated by motif A to motif E.

Two viral contigs were similar to viruses in a new proposed family *Ambiguiviridae*. The family *Ambiguiviridae* contained viruses closely related to members of the genus *Umbravirus* in the family *Tombusviridae*, as well as the (+) ssRNA viruses from insects (Gilbert et al., 2019). All the two viruses, with sizes of 4568 bp and 3380 bp, respectively, contained two ORFs, with the second ORF encoded an RdRp. They were named as AdULV1 and AdULV2, respectively (**Figure 3A**). BLASTp searches showed that the RdRp of AdULV1 shared 54.49% to 70.16% identities with the RdRps of some unclassified viruses, liking Plasmopara viticola lesion associated ambiguivirus 1 (Chiapello et al., 2020) and Erysiphe necator associated ambiguivirus 3. AdULV2 showed the maximal 48.09% identity with Macrophomina phaseolina umbra-like virus 1 (Wang et al., 2021), followed by Erysiphe necator umbra-like virus 2, with 45.24% identity. Phylogenetic analysis based on the RdRps domain of AdULV1 and AdULV2 and other selected viruses showed that AdULV1 and AdULV2 were clustered with members of the proposed family *Ambiguiviridae* (**Figure 3B**). Traditionally, most (+) ssRNA viruses have a GDD at motif C, while this two mycoviruses and selected viruses have a GDN that was previously found in some (-) ssRNA viruses, the dsRNA polymycoviruses and (+)ssRNA hadalkaviruses (Kanhayuwa et al., 2015; Sato et al., 2020) (**Figure 3C**). This shift from GDD to GDN had been proved to adversely affect the enzymatic activity (Poch et al., 1989; Jablonski and Morrow, 1995; Lohmann et al., 1997; Vazquez et al., 2000). According to all of the characteristics, the two viruses might be considered to represent new viral species in the novel proposed family Ambiguiviridae of (+) ssRNA class.

### A novel virus in the family *Totiviridae*

**Figure 4:**
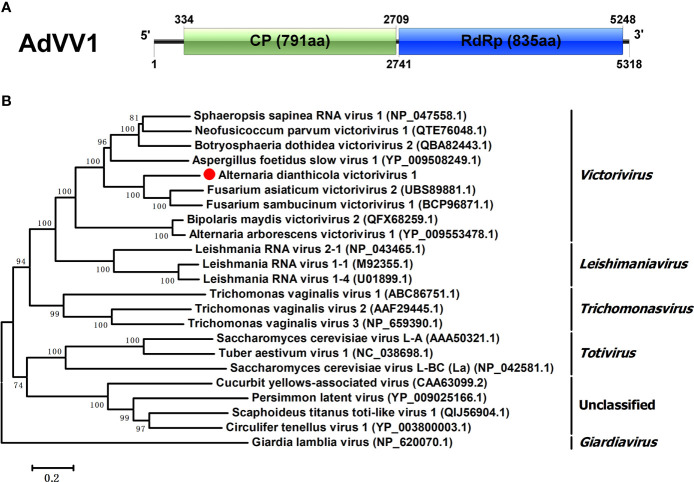
Genomic organization and phylogenetic position of Alternaria dianthicola victorivirus 1 (AdVV1). **(A)** Genome size and organization of AdVV1. The genome of AdVV1 possessed two ORFs, putatively encoding capsid protein and RdRp, respectively, which have been indicated by rectangular boxes. **(B)** Phylogenetic tree was generated based on the RdRp using the NJ method in MEGA 6. Bootstrap values were obtained by 1000 replicates. The scale bar at the lower represented the genetic distance.

The family *Totiviridae* had five genera, namely *Victorivirus*, *Giardiavirus*, *Leishmaniavirus*, *Trichomonasvirus* and *Totivirus* (Ghabrial et al., 2015). Viruses in this family are characterized by linear dsRNA genomes of 4.6-7.0 kbp, which contain two ORFs encoding CP and RdRp or RdRp domain of a fusion protein (Wickner, 2012). One viral sequence, in the length of 5318 bp, was similar to members of the family *Totiviridae*, and was named AdVV1. AdVV1 had two separate ORFs, encoding CP and RdRp of 791 aa and 835 aa, respectively. The 5’ and 3’ untranslated regions (UTRs) of AdVV1 were 333 bp and 70 bp, respectively (**Figure 4A**). Both the CP and RdRp of AdVV1 showed the maximal identities of 63.79% and 58.50% to the corresponding proteins of Fusarium asiaticum victorivirus 2 (FaVV2). It is worth noting that the ORF2 and ORF1 were not overlapped in AdVV1. It is known that the ORFs of viruses in the genus *Victorivirus* were commonly overlapped by the terranucleotide AUGA or pentanucleotide UAAUG, the typical characteristic of the coupled termination-reinitation strategy for translation of downstream ORF in victoriviruses (Li et al., 2011; Zisis et al., 2013). Multiple aa sequences alignment showed that the CP of AdVV1 has an Alanine-Glycine-Proline-rich region on its C-terminal region as reported for victoriviruses, and the RdRp contained eight motifs conserved in the genus *Victorivirus* (**Figure S1**). We performed phylogenetic analysis based on the RdRps of AdVV1 and other selected viruses and revealed that AdVV1 was a new virus, which aggregated with members of the genus *Victorivirus* in the family *Totiviridae* (**Figure 4B**).

### A novel virus in the family *Partitiviridae*

Viruses in family *Partitiviridae* are characterized by small and isometric viruses with bisegmented dsRNA genomes, ranging from 1.4 to 2.4 kbp in size (Liang et al., 2021). The two dsRNA segments separately encode an RdRp and a CP, respectively. However, in addition to RdRp and CP coding sequences, genome segments encoding unknown proteins, such as satellites or defective RNAs, also exist in *partitivirridae*. Up to now, there are seven known or proposed genera in this family, namely *Alphapartitivirus*, *Betapartitivirus*, *Gammapartitivirus*, *Deltapartitivirus*, *Cryspovirus*, *Epsilonpartitivirus* and *Zetapartitivirus* (Vainio et al., 2018; Gilbert et al., 2019).

**Figure 5:**
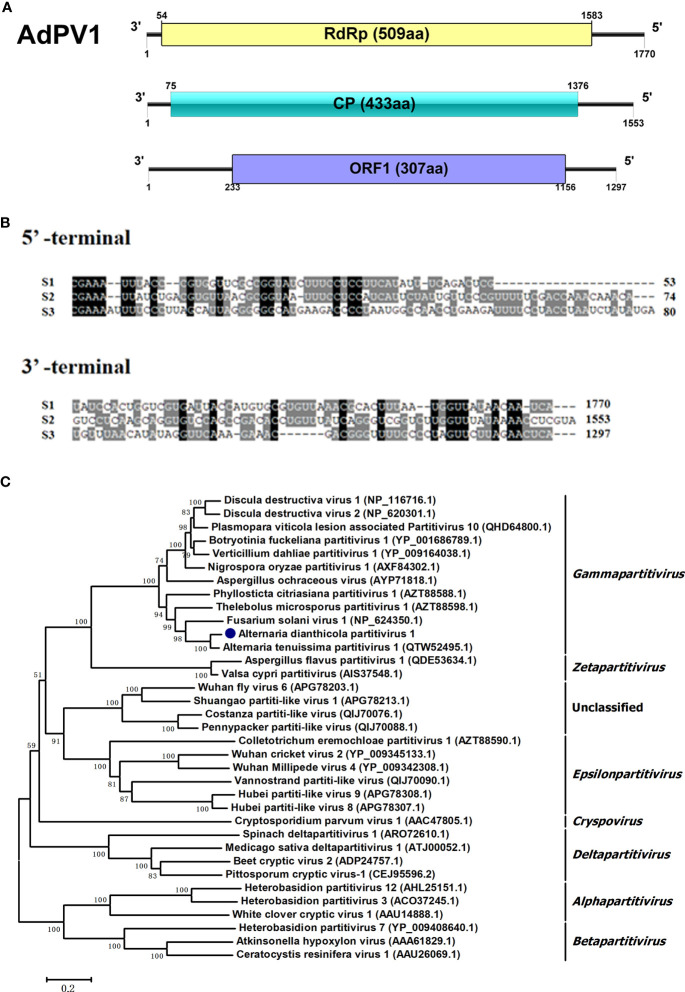
Genome organization and phylogenetic placement of Alternaria dianthicola partitivirus 1 (AdPV1). **(A)** A schematic diagram of the genome organization of AdPV1. The genome of AdPV1 contained three dsRNA segments, with each one has a single ORF, encoding a RdRp, CP and a protein with unknow function, respectively. **(B)** Multiple alignments of 5’ and 3’-terminal nucleotide sequences of the AdPV1 genomic segments. **(C)** NJ phylogenetic tree was constructed based on the RdRp aa sequences.

In this study, virome analysis of strain HNSZ-1 revealed the presence of three viral sequences, in the lengths of 1770 bp, 1553 bp and 1297 bp, which might represent the genome of a novel virus, designated AdPV1(**Figure 5A**). Each of the segments consist of a single ORF encoding a 57.78 kDa, 46.16 kDa and 35.11 kDa protein, respectively. BLASTp homology search revealed that the 57.78 kDa protein was similar to the RdRp of Alternaria tenussima partitivirus 1 with 94.26% identity (AttPV1) (Liang et al., 2021). The 46.16 kDa protein was 96.30% and 85% identical to the CP of Plasmopara viticola lesion associated partitivirus 10 (PVaPV10) (Chiapello et al., 2020) and AttPV1, respectively. The 35.11 kDa protein was similar to the hypothetical protein of PVaPV10 with 78.90% identity. A multiple nucleotide sequence alignment of the three viral segments revealed the presence of conserved sequences “CGAAAUU” at their 5’ UTRs, and high sequence identity at their 3’ UTRs (**Figure 5B**). Therefore, it is speculated that these three RNA fragments might be the same virus genome, according to the sequence homology search and non-coding regions alignment. Phylogenetic analysis based on the RdRp domain suggested that AdPV1 was most likely to be a novel virus related to *Gammapartitivirus* in the family *Partitivirridae* (**Figure 5C**).

### A novel virus in the family *Mymonaviridae*

Viruses in *Mymonaviridae* typically have a linear (-) ssRNA genome, about 10 kbp in size, and enveloped by filamentous virions. Sclerotinia sclerotiorum negative-stranded RNA virus 1 (SsNSRV-1) that was isolated from the devastating plant pathogen fungus *Sclerotinia sclerotiorum* is the first reported (-) ssRNA mycovirus and subsequently be affiliated with the *Mymonaviridae* family (Liu et al., 2014; Amarasinghe et al., 2017; Jiang et al., 2019; Vainio and Suzuki, 2020).

**Figure 6:**
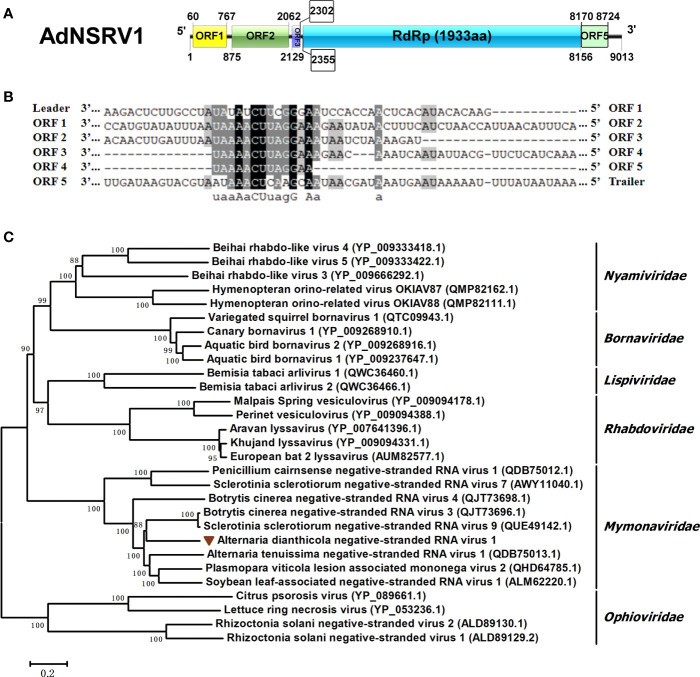
Characterization of the genome structure and phylogenetic analysis of Alternaria dianthicola negative-stranded RNA virus 1 (AdNSRV1). **(A)** Genome size and organization of AdNSRV1. Five ORFs were predicted in the AdNSRV1 genome, which were indicated by the rectangular boxes. **(B)** Comparison of putative gene junction regions between the ORFs in AdNSRV1 shown in 3’- to -5’ orientation. Conserved sequences are highlighted in shade. **(C)** Phylogenetic analysis of AdNSRV1. A phylogenetic tree based on aa alignments of L proteins was constructed, using the NJ method. The virus AdNSRV1 was represented in the phylogenetic tree.

One viral sequence, designated AdNSRV1, was identified in this study. The full length of the AdNSRV1 genome was 9013 nts, with five nonoverlapping ORFs encoding proteins of 235, 395, 57, 1933 and 184 aa, respectively (**Figure 6A**). The 5’- and 3’-UTR were 79 nt and 269 nt long, respectively. Multiple alignment of nucleotide sequence between these ORFs found a semi-conserved sequence, “UAAAACUUAGGAA”, occurred downstream of each ORFs and upstream of ORF1, which was a common feature of Mononegaviruses (**Figure 6B**). These regions might most likely be the gene-junction regions in AdNSRV1 genome and were broadly similar to sequences found in other mononegaviruses (Pringle and Easton, 1997; Wang et al., 2018). BLAST analysis of AdNSRV1 revealed that, among the five ORFs, the largest ORF4 contained conserved regions corresponding to motif pfam00946 (Mononegavirales RNA dependent RNA polymerase; aa sites 238-1040; e-value 2.92e-75) and was 56.03% identical to the RdRp of Sclerotinia sclerotiorum negative-stranded RNA virus 3-U; The ORF2 showed sequence identity with the putative nucleocapsid protein of Plasmopara viticola lesion associated mononega virus 2 (PvLamV2) (Chiapello et al., 2020) and Cryphonectria parasitica sclerotimonavirus 1. ORF1 and ORF5 were predicted to encode hypothetical proteins showing a low level of sequence identity to other members of *Mymonaviridae*. The ORF3 showed no similarity with any other viruses in the NCBI database.

Phylogenetic analysis was performed by using neighbor joining (NJ) model. Phylogenetic tree was constructed based on multiple amino acid sequence alignment of the RdRps of AdNSRV1 and other selected mononegaviruses, including species from each genus within the *Bornaviridae*, *Mymonaviridae*, *Nyamiviridae*, *Rhabdoviridae*, *Lispiviridae* families, and an out group from *Ophioviridae*. The result indicated that AdNSRV1 was clustered with the *Mymonaviridae* family, thus it might be predicted as a novel member in this family (**Figure 6C**).

### Virus elimination, horizontal transmission and effects of viruses infected on HNSZ-1

**Figure 7:**
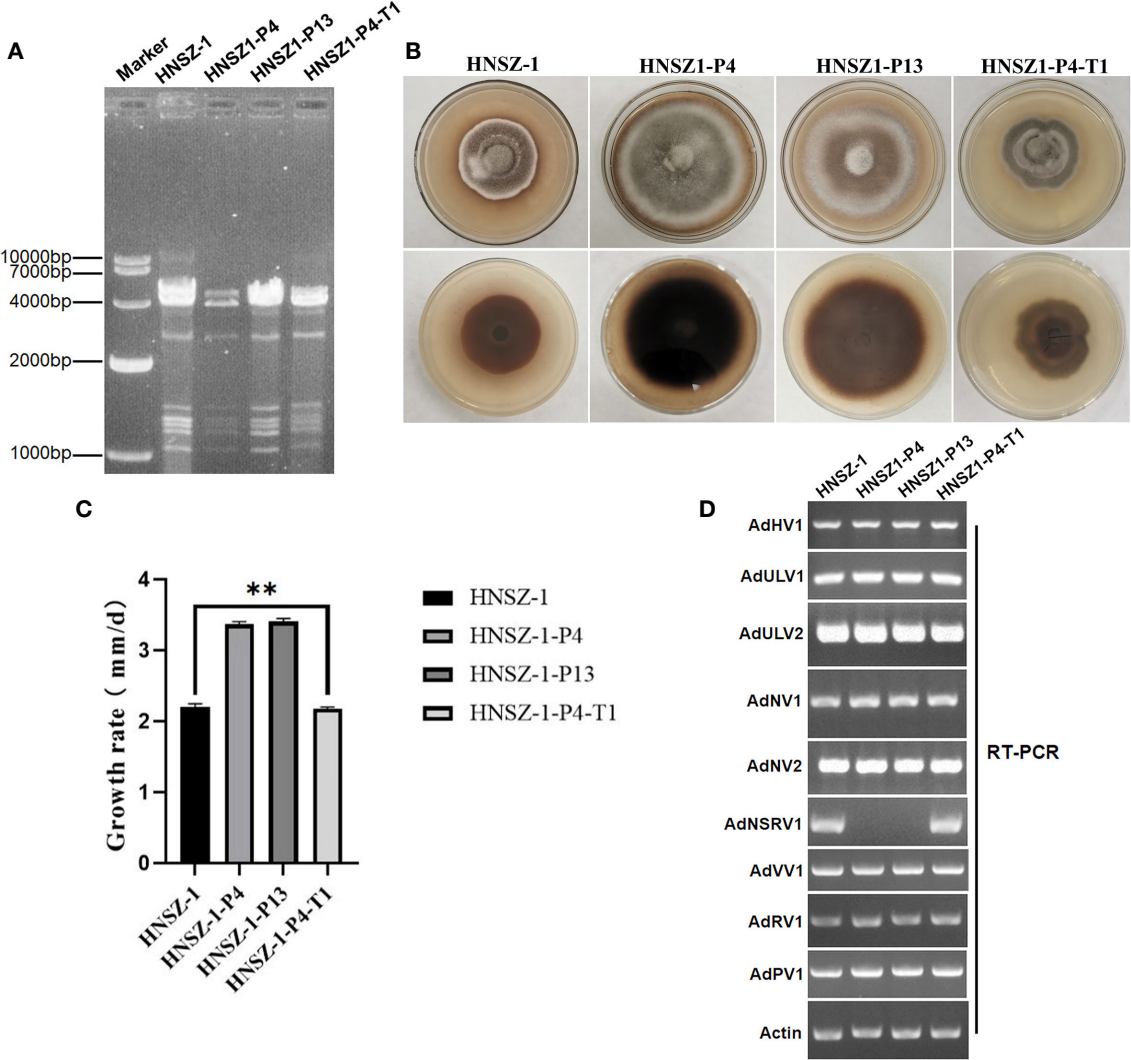
Mycovirus content and biological features of strain HNSZ-1 and other derivative strains. **(A)** Detection of viruses in strain HNSZ-1, its protoplast regeneration derivatives HNSZ-1-P4 and HNSZ-1-P13, and the re-infected derivative strain HNSZ1-P4-T1 by dsRNA extraction. Strain HNSZ-1 and HNSZ1-P4-T1were the original and re-infected strains, respectively, that were co-infected by all the nine viruses. Strains HNSZ-P4 and HNSZ-1-P13 were detected to lack the AdNSRV1. **(B, C)** Colony morphology **(B)** and growth rate ** means significant difference at P < 0.05. **(C)** of strain HNSZ-1, the two protoplast regeneration derivatives HNSZ-1-P4 and HNSZ-1-P13, and the re-infected strain HNSZ1-P4-T1. **(D)** Detection of viruses in strain HNSZ-1, its protoplast regeneration derivatives HNSZ-1-P4 and HNSZ-1-P13, and the re-infected strain HNSZ1-P4-T1 by RT-PCR. Strain HNSZ-1 was the original strain co-infected by nine viruses, while strains HNSZ-P4 and HNSZ-1-P13 were detected to lack the AdNSRV1. Strain HNSZ1-P4-T1 was a derivative strain of HNSZ1-P4 obtained by virus horizontal transmission experiment, that was re-infected by nine viruses including AdNSRV1.

In order to check the possible effects of the viruses on the host fungus, we used protoplast regeneration method for virus curing. After dsRNA extraction and RT-PCR detection, two single-protoplast regeneration derivatives, HNSZ1-P4 and HNSZ1-P13, were obtained, and confirmed to lack the AdNSRV1 (**Figure 7A, C**). In addition, in order to re-introduce AdNSRV1 to the AdNSRV1-curing strain, virus horizontal transmission experiments were carried out using HNSZ1 and HNSZ1-P4 as the donor and recipient of AdNSRV1, respectively. The HNSZ1-P4 derivative strain HNSZ1-P4-T1 re-infected by AdNSRV1 was obtained.

Although no virus-free and other strains carrying different virus combination was obtained. The growth rate and morphology were compared between the original strain HNSZ-1, the two derivative subcultures HNSZ1-P4 and HNSZ1-P13, and the re-infected HNSZ1-P4-T1. On PDA, strains HNSZ1-P4 and HNSZ1-P13 showed regular colony margin as compared to the original strain HNSZ-1 and the re-infected strain HNSZ1-P4-T1 (**Figure 7B**). The average growth rate of strain HNSZ-1 and HNSZ1-P4-T1 were significantly lower than that of HNSZ1-P4 and HNSZ1-P13, but no significant difference was exhibited between the HNSZ1-P4 and HNSZ1-P13 (**Figure 7D**). These results indicated that AdNSRV1 in strain HNSZ-1 might be involved in the phenotypic alteration of the host fungus. However, the effects of each virus on the host fungus or any interactions between these co-infected viruses remain warrant further exploration.

## Conclusions

In this study, we characterized the virome of an *A. dianthicola* strain HNSZ-1, that was isolated from the diseased leaves of *D.chinensis.* After dsRNA extraction, high-throughput sequencing, RT-PCR detection and conventional Sanger sequencing, the complete genome of nine viruses co-infected in strain HNSZ-1 were obtained. Genome organization, homology search and phylogenetic analysis revealed that, among the nine viruses, a dsRNA virus named AdRV1 has been identified to be an unclassified virus in our previously study (Hu et al., 2020), and five viruses named AdHV1, AdULV1, AdULV2, AdNSRV1 and AdVV1 were predicted to represent new mycovirus species belonging to the families *Hypoviridae*, *Ambiguiviridae*, *Mymonaviridae* and *Totiviridae*, respectively. Meanwhile, viruses AdNV1, AdNV2 and AdPV1 have more than 95% similarity to the viral sequences in other fungal species in the NCBI database. Thus, they might be new strains of these previously recorded mycoviruses, which were assigned to the families *Narnaviridae* and *Partitiviridae*, respectively. Thus far, the identification of co-infection of mycoviryses in this study enriched the diversity of virus constitution in *Alternaria*.

The application of high-throughput sequencing technology for virus hunting has greatly promoted the development of virology in recent years that accelerated the discovery of virus diversity, origin and evolution from various materials including animals, plants, fungi and environment (Adams et al., 2009; Roossinck et al., 2015; Marzano et al., 2016; Shi et al., 2016; Chiapello et al., 2020; Myers et al., 2020; Wang et al., 2021; Ruiz-Padilla et al., 2021; Kondo et al., 2022; Dominguez-Huerta et al., 2022). For mycoviruses, many studies have reported that the co-infection of a large number of viruses in single fungal strains is a common phenomenon, which usually appeared in the necrotrophic fungi *R. solani* and *S. sclerotiorum* (Bartholomäus et al., 2016; Mu et al., 2021). Although a number of viruses have been reported in *Alternaria* species, no strain has been infected by comparable number of virus species as has been identified in this study. Therefore, we speculate that there are a large number of mycoviral resources in *Alternaria*, given that the *Alternaria* is a large taxonomic fungal group. Our study is a step forward the screening of mycoviruses infecting *Alternaria* species.

In this study, we obtained two protoplast regeneration derivatives, lacking the AdNSRV1 infection, and found that AdNSRV1 might be related to growth rate and morphology in the host *A. dianthicola.* This was confirmed by virus elimination and horizontal transmission experiments. Of course, the direct evidence that AdNSRV1 had explicit effects on its host remain limited. Further efforts including virions transfection of AdNSRV1 to the virus-free strains were also warranted. In addition, we also conducted the virus horizontal transmission experiments in order to transfer AdNSRV1 singly from HNSZ-1 to other virus-free strains with the same genetic background, but failed. It is possible that other viruses co-infecting with AdNSRV1 might have potential interactions with AdNSRV1, as have been inferred by previously reports (Li et al., 2022a; Li et al., 2022b). Since we have not obtained other derivative strains infected by each virus alone or by different virus combination, the possible effects of each virus on its host fungus, or whether there is any interaction between these co-infected viruses, remains to be elucidated. In conclusion, to our knowledge, this is the first report for virome characterization of *A. dianthus*. This study might provide an important insight for understanding the diversity and evolutionary of mycoviruses and screening mycoviruses for biological control. To a certain extent, it was also conducive to study the interactions between viruses or viruses and their fungal host.

## Data availability statement

The datasets presented in this study can be found in online repositories. The names of the repository/repositories and accession number(s) can be found in the article/**Supplementary Material**.

## Author contributions

JZ, PL, and SZ: sampling, investigation, writing-original draft. JZZ and XL: investigation, results interpretation, reviewing and editing original draft. JZ and BG: conceptualization, supervision, results interpretation, writing-reviewing and editing- original draft. All authors contributed to the article and approved the submitted version.

## Funding

This research was supported by the Key Foundation of Hunan Educational Committee (Grant No. 20A257); the Hunan Innovation and entrepreneurship training Foundation for Undergraduates (grant no. S202110537045); the Hunan Provincial Innovation Foundation for Postgraduates (grant no. CX20210676).

## Conflict of interest

The authors declare that the research was conducted in the absence of any commercial or financial relationships that could be construed as a potential conflict of interest.

## Publisher’s note

All claims expressed in this article are solely those of the authors and do not necessarily represent those of their affiliated organizations, or those of the publisher, the editors and the reviewers. Any product that may be evaluated in this article, or claim that may be made by its manufacturer, is not guaranteed or endorsed by the publisher.

## References

[B1] Adams I. P. Glover R. H. Monger W. A. Mumford R. Jackeviciene E. Navalinskiene M.. (2009). Next-generation sequencing and metagenomic analysis: A universal diagnostic tool in plant virology. Mol. Plant Pathol. 10, 537–545. doi: 10.1111/j.1364-3703.2009.00545.x 19523106 PMC6640393

[B2] Amarasinghe G. K. Bào Y. Basler C. F. Bavari S. Beer M. Bejerman N.. (2017). Taxonomy of the order *Mononegavirales*: update 2017. Arch. Virol. 162, 2493–2504. doi: 10.1007/s00705-017-3311-7 28389807 PMC5831667

[B3] Ambra P. Davide S. Angelo G. Maria L. G. (2013). A new method for detection of five *alternaria* toxins in food matrices based on LC-APCI-MS. Food Chem. 140, 161–167. doi: 10.1016/j.foodchem.2012.12.065 23578628

[B4] Aulia A. Hyodo K. Hisano S. Kondo H. Hillman B. I. Suzuki N. (2021). Identification of an RNA silencing suppressor encoded by a symptomless fungal hypovirus, cryphonectria hypovirus 4. Biology 10, 100. doi: 10.3390/biology10020100 33572564 PMC7912522

[B5] Bartholomäus A. Wibberg D. Winkler A. Pühler A. Schlüter A. Varrelmann M. (2016). Deep sequencing analysis reveals the mycoviral diversity of the virome of an avirulent isolate of *Rhizoctonia solani* AG-2-2 IV. PloS One 11, e0165965. doi: 10.1371/journal.pone.0165965 27814394 PMC5096721

[B6] Chiapello M. RodríguezRomero J. Ayllón M. A. Turina M. (2020). Analysis of the virome associated to grapevine downy mildew lesions reveals new mycovirus lineages. Virus Evol. 6, veaa058. doi: 10.1093/ve/veaa058 33324489 PMC7724247

[B7] Daniela L. Jasmin N. D. Bradley I. H. (2005). Genome analysis of cryphonectria hypovirus 4, the most common hypovirus species in north America. Virology 337, 192–203. doi: 10.1016/j.virol.2005.03.038 15914232

[B8] Das S. Alam M. M. Zhang R. Hisano S. Suzuki N. (2021). Proof of concept of the yadokari nature: a capsidless replicase-encoding but replication-dependent positive-sense single-stranded RNA virus hosted by an unrelated double-stranded RNA virus. J. Virol. 95, e00467–e00421. doi: 10.1128/JVI.00467-21 PMC8356704 34106772

[B9] Dominguez-Huerta G. Zayed A. A. Wainaina J. M. Guo J. Tian F. Pratama A. A.. (2022). Diversity and ecological footprint of global ocean RNA viruses. Science 376 (6598), 1202–1208. doi: 10.1126/science.abn6358 35679415

[B10] Duan C. F. Long Y. Q. Chen H. Yang G. H. Gui M. Liu G. H. (2015). First report of *Alternaria dianthicola* causing fower blight on carnation in China. EPPO Bull. 45, 195–198. doi: 10.1111/epp.12200

[B11] García-Pedrajas M. D. Cañizares M. C. Sarmiento-Villamil J. L. Jacquat A. G. Dambolena J. S. (2019). Mycoviruses in biological control: From basic research to field implementation. Phytopathology 109, 1828–1839. doi: 10.1094/PHYTO-05-19-0166-RVW 31398087

[B12] Ghabrial S. A. Caston J. R. Jiang D. H. Nibert M. L. Suzuki N. (2015). 50-plus years of fungal viruses. Virology 479, 356–368. doi: 10.1016/j.virol.2015.02.034 25771805

[B13] Ghabrial S. A. Suzuki N. (2009). Viruses of plant pathogenic fungi. Annu. Rev. Phytopathol. 47, 353–384. doi: 10.1146/annurev-phyto-080508-081932 19400634

[B14] Gilbert K. B. Holcomb E. E. Allscheid R. L. Carrington J. C. (2019). Hiding in plain sight: New virus genomes discovered *via* a systematic analysis of fungal public transcriptomes. PloS One 14, e0219207. doi: 10.1371/journal.pone.0219207 31339899 PMC6655640

[B15] Grabherr M. G. Haas B. J. Yassour M. Levin J. Z. Thompson D. A. Amit I.. (2011). Full-length transcriptome assembly from RNA-seq data without a reference genome. Nat. Biotechnol. 29, 644–652. doi: 10.1038/nbt.1883 21572440 PMC3571712

[B16] Hao F. Ding T. Wu M. Zhang J. Yang L. Chen W.. (2018). Two novel hypovirulence-associated mycoviruses in the phytopathogenic fungus *Botrytis cinerea*: Molecular characterization and suppression of infection cushion formation. Viruses 10, 254. doi: 10.3390/v10050254 PMC5977247 29757259

[B17] Hajime Y. Satoko K. Tsutae I. (2012). Molecular characterization of a new hypovirus infecting a phytopathogenic fungus, *Valsa ceratosperma* . Virus Res. 165, 143–150. doi: 10.1016/j.virusres.2012.02.008 22366520

[B18] Hillman B. I. Annisa A. Suzuki N. (2018). Viruses of plant-interacting fungi. Adv. Virus Res. 100, 99–116. doi: 10.1016/bs.aivir.2017.10.003 29551145

[B19] Hillman B. I. Cai G. H. (2013). The family *narnaviridae*: simplest of RNA viruses. Adv. Virus Res. 86, 149–176. doi: 10.1016/B978-0-12-394315-6.00006-4 23498906

[B20] Hu Z. Guo J. Da Gao B. Zhong J. (2020). A novel mycovirus isolated from the plant-pathogenic fungus *Alternaria dianthicola* . Arch. Virol. 165, 2105–2109. doi: 10.1007/s00705-020-04700-9 32556598

[B21] Jablonski S. A. Morrow C. D. (1995). Mutation of the aspartic acid residues of the GDD sequence motif of poliovirus RNA-dependent RNA polymerase results in enzymes with altered metal ion requirements for activity. J. Virol. 69, 1532–1539. doi: 10.1128/jvi.69.3.1532-1539.1995 7853486 PMC188746

[B22] Jia J. Mu F. Fu Y. Cheng J. Lin Y. Li B.. (2022). A capsidless virus is trans-encapsidated by a bisegmented *Botybirnavirus* . J. Virol. 96, e00296–e00222. doi: 10.1128/jvi.00296-22 PMC9093100 35446143

[B23] Jiang D. Ayllón M. A. Marzano S. Y. L. (2019). ICTV virus taxonomy profile: Mymonaviridae. J. Gen. Virol. 100, 1343–1344. doi: 10.1099/jgv.0.001301 31478828 PMC12841598

[B24] Kanhayuwa L. Kotta-Loizou I. Özkan S. Gunning A. P. Coutts R. H. (2015). A novel mycovirus from aspergillus fumigatus contains four unique dsRNAs as its genome and is infectious as dsRNA. Proc. Natl. Acad. Sci. U. S. A. 112, 9100–9105. doi: 10.1073/pnas.1419225112 26139522 PMC4517262

[B25] Kondo H. Botella L. Suzuki N. (2022). Mycovirus diversity and evolution Revealed/Inferred from recent studies. Annu. Rev. Phytopathol. 60, 307–336. doi: 10.1146/annurev-phyto-021621-122122 35609970

[B26] Kondo H. Kanematsu S. Suzuki N. (2013). Viruses of the white root rot fungus, *Rosellinia necatrix* . Adv. Virus Res. 86, 177–214. doi: 10.1016/B978-0-12-394315-6.00007-6 23498907

[B27] Koonin E. V. Choi G. H. Nuss D. L. Shapira R. Carrington J. C. (1991). Evidence for common ancestry of a chestnut blight hypovirulence-associated double-stranded RNA and a group of positive-strand RNA plant viruses. Proc. Natl. Acad. Sci. U.S.A. 88, 10647–10651. doi: 10.1073/pnas.88.23.10647 1961731 PMC52987

[B28] Lau S. K. Lo G. C. Chow F. W. Fan R. Y. Cal J. J. Yen K. Y.. (2018). Novel partitivirus enhances virulence of and causes aberrant gene expression in talaromyces marneffei. mBio 9, e00947–e00918. doi: 10.1128/mBio.00947-18 29895639 PMC6016240

[B29] Liang Z. J. Wang X. Y. Hua H. H. Cao W. Zhou T. Zhao C.. (2021). Full genome sequence of a new three-segment gammapartitivirus from the phytopathogenic fungus *Alternaria tenuissima* on cotton in China. Arch. Virol. 166, 973–976. doi: 10.1007/s00705-020-04937-4 33427965

[B30] Li H. Bian R. L. Liu Q. Yang L. Pang T. X. Salaipeth X.. (2019). Identification of a novel hypovirulence-inducing hypovirus from *Alternaria alternata* . Front. Microbiol. 10, 1076. doi: 10.3389/fmicb.2019.01076 PMC6530530 31156589

[B31] Li B. Cao Y. H. Ji Z. X. Zhang J. Y. Meng X. L. Dai P. B.. (2022a). Coinfection of two mycoviruses confers hypovirulence and reduces the production of mycotoxin alternariol in *Alternaria alternata* f. sp. *mali* . Front. Microbiol. 13, 910712. doi: 10.3389/fmicb.2022.910712 35756001 PMC9218907

[B32] Li H. Havens W. M. Nibert M. L. Ghabrial S. A. (2011). RNA Sequence determinants of a coupled termination-reinitiation strategy for downstream open reading frame translation in helminthosporium victoriae virus 190S and other *victoriviruses* (Family *Totiviridae*). J. Virol. 85 (14), 7343–7352. doi: 10.1128/JVI.00364-11 21543470 PMC3126583

[B33] Li Y. Y. Lyu R. L. Hai D. Jia J. C. Jiang D. H. Fu Y. P.. (2022b). Two novel rhabdoviruses related to hypervirulence in a phytopathogenic fungus. J. Virol. 96, e00012–e00022. doi: 10.1128/jvi.00012-22 PMC9044937 35389267

[B34] Liu L. J. Xie J. T. Cheng J. S. Fu Y. P. Li G. Q. Yi X.. (2014). Fungal negative-stranded RNA virus that is related to bornaviruses and nyaviruses. Proc. Natl. Acad. Sci. U.S.A. 111, 12205–12210. doi: 10.1073/pnas.1401786111 25092337 PMC4143027

[B35] Lohmann V. Korner F. Herian U. Bartenschlager R. (1997). Biochemical properties of hepatitis c virus NS5B RNA-dependent RNA polymerase and identification of amino acid sequence motifs essential for enzymatic activity. J. Virol. 71, 8416–8428. doi: 10.1128/jvi.71.11.8416-8428.1997 9343198 PMC192304

[B36] Mahmoud E. K. Michael N. P. (2014). Characterisation of a novel hypovirus from *Sclerotinia sclerotiorum* potentially representing a new genus within the *Hypoviridae* . Virology 464, 441–449. doi: 10.1016/j.virol.2014.07.005 25108682

[B37] Maiti C. K. Sen S. Paul A. K. Acharya K. (2007). First report of *Alternaria dianthicola* causing leaf blight on *Withania somnifera* from India. Plant Dis. 464, 441–449. doi: 10.1016/j.virol.2014.07.005 30781215

[B38] Marzano L. S. Nelson B. D. Ajayi-Oyetunde O. Bradley C. A. Hughes T. J. Hartman G. L.. (2016). Identification of diverse mycoviruses through metatranscriptomics characterization of the viromes of five major fungal plant pathogens. J. Virol. 90, 6846–6863. doi: 10.1128/JVI.00357-16 27194764 PMC4944287

[B39] Ma G. P. Zhang X. F. Hua H. H. Zhou T. Wu X. H. (2020). Molecular and biological characterization of a novel strain of alternaria alternata chrysovirus 1 identified from the pathogen *Alternaria tenuissima* causing watermelon leaf blight. Virus Res. 280, 197904. doi: 10.1016/j.virusres.2020.197904 32105762

[B40] Mizutani Y. Uesaka K. Ota A. Calassanzio M. Ratti C. Suzuki T.. (2021). *De novo* sequencing of novel mycoviruses from *Fusarium sambucinum*: an attempt on direct RNA sequencing of viral dsRNAs. Front. Microbiol. 12, 641484. doi: 10.3389/fmicb.2021.641484 33927702 PMC8076516

[B41] Morris T. J. Dodds J. A. (1979). Isolation and analysis of double stranded RNA from virus-infected plant and fungal tissue. Phytopathology 69, 854–858. doi: 10.1094/Phyto-69-854 40934407

[B42] Mu F. Li B. Cheng S. Jia J. Jiang D. Fu Y.. (2021). Nine viruses from eight lineages exhibiting new evolutionary modes that co-infect a hypovirulent phytopathogenic fungus. PloS Pathog. 17, e1009823. doi: 10.1371/journal.ppat.1009823 34428260 PMC8415603

[B43] Myers J. M. Bonds A. E. Clemons R. A. Thapa N. A. Simmons D. R. Carter-House D.. (2020). Survey of early-diverging lineages of fungi reveals abundant and diverse mycoviruses. mBio 11, e02027–e02020. doi: 10.1128/mBio.02027-20 32900807 PMC7482067

[B44] Okada R. Ichinose S. Takeshita K. Urayama S. I. Fukuhara T. Komatsu K.. (2018). Molecular characterization of a novel mycovirus in Alternaria alternata manifesting two-sided effects: Down-regulation of host growth and up-regulation of host plant pathogenicity. Virology 519, 23–32. doi: 10.1016/j.virol.2018.03.027 29631173

[B45] Nuss D. L. (2005). Hypovirulence: mycoviruses at the fungal-plant interface. Nat. Rev. Microbiol. 3, 632–642. doi: 10.1038/nrmicro1206 16064055

[B46] Pearson M. N. Beever R. E. Boine B. Arthur K. (2009). Mycoviruses of flamentous fungi and their relevance to plant pathology. Mol. Plant Pathol. 10, 115–128. doi: 10.1111/j.1364-3703.2008.00503.x 19161358 PMC6640375

[B47] Poch O. Sauvaget I. Delarue M. Tordo N. (1989). Identification of four conserved motifs among the RNA dependent polymerase encoding elements. EMBO J. 8, 3867–3874. doi: 10.1002/j.1460-2075.1989.tb08565.x 2555175 PMC402075

[B48] Pringle C. R. Easton A. J. (1997). Monopartite negative strand RNA genomes. Semin Virol. 8, 49–57. doi: 10.1006/smvy.1997.0105

[B49] Rigling D. Prospero S. (2018). *Cryphonectria parasitica*, the causal agent of chestnut blight: invasion history, population biology and disease control. Mol. Plant Pathol. 19, 7–20. doi: 10.1111/mpp.12542 28142223 PMC6638123

[B50] Roossinck M. J. Martin D. P. Roumagnac P. (2015). Plant virus metagenomics: advances in virus discovery. Phytopathology 105, 716–727. doi: 10.1094/PHYTO-12-14-0356-RVW 26056847

[B51] Ruiz-Padilla A. RodríguezRomero J. GómezCid I. Pacifico D. Ayllón M. A. (2021). Novel mycoviruses discovered in the mycovirome of a necrotrophic fungus. mBio 12, e03705–e03720. doi: 10.1128/mBio.03705-20 33975945 PMC8262958

[B52] Sato Y. Shamsi W. Jamal A. Bhatti M. F. Kondo H. Suzuki N. (2020). Hadaka virus 1: A capsidless eleven-segmented positive-sense single-stranded RNA virus from a phytopathogenic fungus, *Fusarium oxysporum* . mBio 11, e00450–e00420. doi: 10.1128/mBio.00450-20 32457242 PMC7251205

[B53] Shi M. Lin X. D. Tian J. H. Chen L. J. Chen X. Li C. X.. (2016). Redefining the invertebrate RNA virosphere. Nature 540, 539–543. doi: 10.1038/nature20167 27880757

[B54] Suzuki N. Ghabrial S. A. Kim K. H. Pearson M. Marzano S. Y. L. Yaegashi H.. (2018). ICTV virus taxonomy profile. Hypoviridae. J. Gen. Virol. 99, 615–616. doi: 10.1099/jgv.0.001055 29589826 PMC12662185

[B55] Tamura K. Stecher G. Peterson D. Filipski A. Kumar S. (2013). MEGA6: molecular evolutionary genetics analysis version 6.0. Mol. Biol. Evol. 30, 2725–2729. doi: 10.1093/molbev/mst197 24132122 PMC3840312

[B56] Thapa V. Roossinck M. J. (2019). Determinants of coinfection in the mycoviruses. Front. Cell Infect. Mi. 9, 169. doi: 10.3389/fcimb.2019.00169 PMC6542947 31179246

[B57] Thompson J. D. Gibson T. J. Plewniak F. Jeanmougin F. Higgins D. G. (1997). The CLUSTAL_X windows interface: Fexible strategies for multiple sequence alignment aided by quality analysis tools. Nucleic Acids Res. 25, 4876–4882. doi: 10.1093/nar/25.24.4876 9396791 PMC147148

[B58] Vainio E. J. Chiba S. Ghabrial S. A. Maiss E. Roossinck M. Sabanadzovic S.. (2018). ICTV virus taxonomy profile: *Partitiviridae* . J. Gen. Virol. 99, 17–18. doi: 10.1099/jgv.0.000985 29214972 PMC5882087

[B59] Vainio E. J. Suzuki N. (2020). Mixed infection by a partitivirus and a negative-sense RNA virus related to mymonaviruses in the polypore fungus *Bondarzewia berkeleyi* . Virus Res. 286, 198079. doi: 10.1016/j.virusres.2020.198079 32599089

[B60] Vazquez A. L. Alonso J. M. Parra F. (2000). Mutation analysis of the GDD sequence motif of a calicivirus RNA dependent RNA polymerase. J. Virol. 74, 3888–3891. doi: 10.1128/JVI.74.8.3888-3891.2000 10729164 PMC111898

[B61] Vijaykrishna D. Mukerji R. Smith G. J. (2015). RNA Virus reassortment: an evolutionary mechanism for host jumps and immune evasion. PloS Pathog. 11, e1004902. doi: 10.1371/journal.ppat.1004902 26158697 PMC4497687

[B62] Wang L. He H. Wang S. C. Chen X. G. Qiu D. W. Kondo H.. (2018). Evidence for a novel negative-stranded RNA mycovirus isolated from the plant pathogenic fungus *Fusarium graminearum* . Virology 518, 232–240. doi: 10.1016/j.virol.2018.03.008 29549785

[B63] Wang J. Ni Y. X. Liu X. T. Zhao H. Xiao Y. N. Xiao X. Q.. (2021). Divergent RNA viruses in *Macrophomina phaseolina* exhibit potential as virocontrol agents. Virus Evol. 7, veaa095. doi: 10.1093/ve/veaa095 33505706 PMC7816680

[B64] Wickner G. (2012). “Family totiviridae,” in Virus taxonomy: Classification and nomenclature of viruses, ninth report of the international committee on taxonomy of viruses. Eds. King A. M. Q. Adams M. J. Carstens E. B. Lefkowitz E. J.(Waltham, MA, USA: Elsevier), 639–650.

[B65] Woudenberg J. H. C. Groenewald J. Z. Binder M. Crous P. W. (2013). *Alternaria* redefined. Stud. Mycol. 75, 171–212. doi: 10.3114/sim0015 24014900 PMC3713888

[B66] Xiang J. Fu M. Hong N. Zhai L. F. Xiao F. Wang G. (2017). Characterization of a novel botybirnavirus isolated from a phytopathogenic alternaria fungus. Arch. Virol. 162, 3907–3911. doi: 10.1007/s00705-017-3543-6 28891001

[B67] Xie J. Jiang D. H. (2014). New insights into mycoviruses and exploration for the biological control of crop fungal diseases. Annu. Rev. Phytopathol. 52, 45–68. doi: 10.1146/annurev-phyto-102313-050222 25001452

[B68] Xie J. Ghabrial S. A. (2012). Molecular characterizations of two mitoviruses co-infecting a hyovirulent isolate of the plant pathogenic fungus Sclerotinia sclerotiorum. Virology 428, 77–85. doi: 10.1016/j.virol.2012.03.015 22520836

[B69] Yang D. Wu M. Zhang J. Chen W. Li G. Yang L.. (2018). Sclerotinia minor endornavirus 1, a novel pathogenicity debilitation-associated mycovirus with a wide spectrum of horizontal transmissibility. Viruses 10, 589. doi: 10.3390/v10110589 PMC6266790 30373273

[B70] Yu X. Li B. Fu Y. Jiang D. Ghabrial S. A. Li G. Q.. (2010). A geminivirus-related DNA mycovirus that confers hypovirulence to a plant pathogenic fungus. Proc. Natl. Acad. Sci. U.S.A. 107, 8387–8392. doi: 10.1073/pnas.0913535107 20404139 PMC2889581

[B71] Zhang R. Hisano S. Tani A. Kondo H. Kanematsu S. Suzuki N. (2016). A capsidless ssRNA virus hosted by an unrelated dsRNA virus. Nat. Microbiol. 1, 1–6. doi: 10.1038/nmicrobiol.2015.1 27571749

[B72] Zhang H. X. Xie J. T. Fu Y. P. Cheng J. S. Qu Z. Zhao Z. Z.. (2020). A 2-kb mycovirus converts a pathogenic fungus into a beneficial endophyte for brassica protection and yield enhancement. Mol. Plant 13, 1420–1433. doi: 10.1016/j.molp.2020.08.016 32998002

[B73] Zhu J. Z. Zhu H. J. Gao B. D. Zhou Q. Zhong J. (2018). Diverse, novel mycoviruses from the virome of a hypovirulent *Sclerotium rolfsii* strain. Front. Plant Sci. 9, 1738. doi: 10.3389/fpls.2018.01738 30542362 PMC6277794

[B74] Zisis K. Noemi H. Robert H. A. C. (2013). The complete nucleotide sequence of a totivirus from *Aspergillus foetidus* . Arch. Virol. 158, 263–266. doi: 10.1007/s00705-012-1368-x 22729614

