# Supplementary material for: Novel and diverse mycoviruses co-infecting a single strain of the phytopathogenic fungus Alternaria dianthicola
Source: Front Cell Infect Microbiol. 2022 Sep 27;12:980970. doi: 10.3389/fcimb.2022.980970 (PMC9552818; doi:10.3389/fcimb.2022.980970)
Supplement: Supplementary file 1 [file DataSheet_1.zip › Figure S1.docx]

Fig.S1


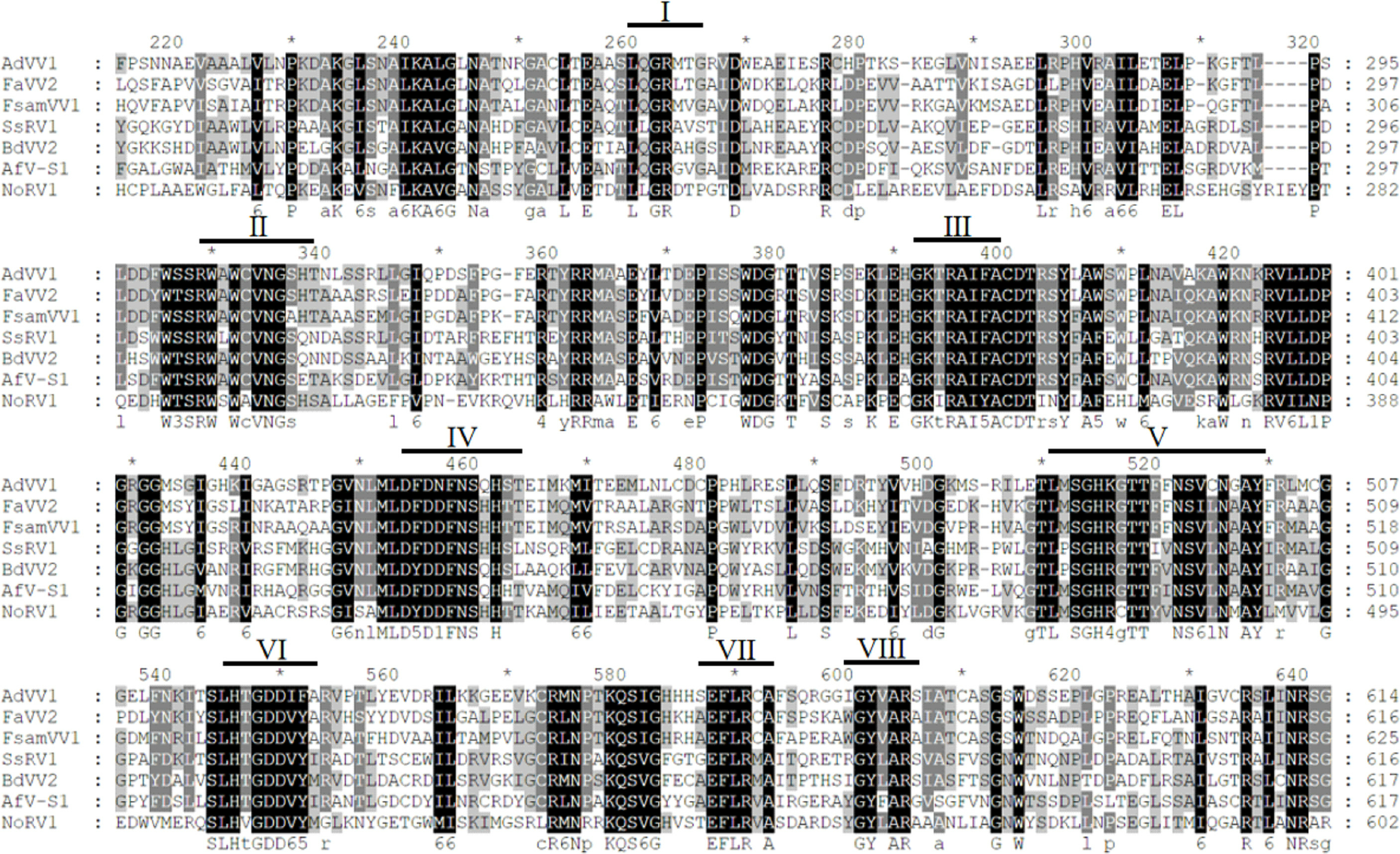


**FIGURE S1** Multiple alignment illustrating conserved motifs in the RdRps of AdVV1 and other similar viruses in the family *Totiviridae.*
